# Food Production and Processing Considerations of Allergenic Food Ingredients: A Review

**DOI:** 10.1155/2012/746125

**Published:** 2011-12-01

**Authors:** Pedro A. Alvarez, Joyce I. Boye

**Affiliations:** Food Research and Development Centre, Agriculture and Agri-Food Canada, 3600 Boulevard Casavant West, Saint-Hyacinthe, QC, Canada J2S 8E3

## Abstract

Although most consumers show no adverse symptoms to food allergens, health consequences for sensitized individuals can be very serious. As a result, the Codex General Standard for the Labelling of Prepackaged Foods has specified a series of allergenic ingredients/substances requiring mandatory declaration when present in processed prepackaged food products. Countries adhering to international standards are required to observe this minimum of eight substances, but additional priority allergens are included in the list in some countries. Enforcement agencies have traditionally focused their effort on surveillance of prepackaged goods, but there is a growing need to apply a bottom-up approach to allergen risk management in food manufacturing starting from primary food processing operations in order to minimize the possibility of allergen contamination in finished products. The present paper aims to review food production considerations that impact allergen risk management, and it is directed mainly to food manufacturers and policy makers. Furthermore, a series of food ingredients and the allergenic fractions identified from them, as well as the current methodology used for detection of these allergenic foods, is provided.

## 1. Introduction

Exposure to undeclared ingredients in processed foods constitutes an important source of concern for allergic individuals. Although the vast majority of consumers will not show any adverse reactions of medical concern, contact with tainted food products could translate to anaphylaxis and potentially death for sensitized individuals. The challenge to find safe ready-to-eat foods is even greater for people displaying multiple food allergies, a phenomenon of particular importance in children. As a result, proper package labelling is enforced on the food manufacturer, and active surveillance for priority allergens on finished goods has constituted one of the primary activities of governmental agencies worldwide.

Adaptations to the modern fast-paced lifestyle have led to increased commercialization of processed prepackaged food products to keep up with people's demand for convenience and variety. Some of the many changes in the way popular foods are produced include greater use of machines to reduce processing times, improve shelf life, and develop superior textural properties, but all of these advancements have also introduced many additional ingredients to the modern industrial recipes for prepackaged foods. New ingredients or processing aids are used to help in machinability of products at intermediate steps of manufacture (e.g., glycerine in cookies). Other new ingredients improve texture of the final product (e.g., soybean flour in sausages [1]) whereas others improve shelf life (e.g., sulphites in dried fruits [2]). Many new ingredients in these complex industrial formulations are known food allergens.

An additional level of complexity is introduced when the purity and authenticity of raw materials is in question. The cascade effect of using a heavily contaminated food ingredient in a complex recipe could be a source of confusion for all parties (manufacturers, consumers, and enforcement agencies) while still posing a threat to the health of consumers. A good example of this was a Margherita pizza recipe made with tomato sauce, mozzarella cheese, basil, and oregano, on a wheat flour base pie (wheat flour, water, bakers' yeast, and salt); although this was a simple pizza recipe judging by the number of ingredients, it was the source of an anaphylactic reaction to buckwheat hidden within the crust dough for a young woman [3]. In some cases the allergen containing ingredient is a small fraction of a formula and the dilution effect of the recipe is enough to protect the consumer, but the threshold dose to trigger clinical symptoms varies greatly and depends on the sensitization level of the individual. For some, multiple oral exposures with a minimum cumulative dosage in the order of grams is required to cause a reaction whereas others require a dosage in only micrograms levels to elicit symptoms [4]. A summary of minimum levels to elicit adverse effects to some allergenic foods can be found in Table 1. The limit of detection and method of commercial test for these allergens is also included in this table.

Regulation regarding which food allergens to consider varies globally, although the current FAO/WHO Codex General Standard for the Labelling of Prepackaged Foods contains a defined list of eight foods or substances and their derivatives [5]. Similarly, Canada currently recognizes nine priority food allergens: peanut, tree nuts, sesame seed, milk, egg, seafood (fish, crustaceans, and shellfish), soy, wheat, and sulphites [6]. The United States of America recognizes soybeans in addition to the allergens in Codex [7, 8]. Australia and New Zealand includes bee products (bee pollen, propolis, and royal jelly) besides the Codex standard [9, 10]. The European Union regulations includes soybeans, celery, mustard, sesame seeds, and lupin in addition to the Codex standard [11]. Japan enforces the labelling of five allergens: wheat, buckwheat, egg, milk, and peanut; but recommends the labelling of another twenty foods: abalone, squid, salmon roe, shrimp, orange, crab, kiwi fruit, beef, walnut, salmon, mackerel, soybean, chicken, pork, matsutake mushroom, peach, yam, apple, gelatin, and banana [12]. The severity of patients' reactions to specific allergens and worldwide or regional incidence of the allergy constitute the general guideline to include allergenic foods on priority lists.

The present paper aims to review certain food production considerations with implications on allergen risk management. Also, a series of food ingredients and the current allergenic fractions identified from them, as well as the methodology for detection of these allergens in foods are reviewed. The collected information is intended to raise awareness for food manufacturing operations as well as to help in policy making.

## 2. Technical and Technological Considerations for Allergen Risk Management

The precautionary statement now widely used in prepackaged foods: “may contain traces of…” arises from a potential risk of allergen contamination which could occur either during manufacturing or due to the presence of allergens in raw materials. Described below are some of the inherent risks of allergen contamination in the food manufacturing process. 

### 2.1. Issues at Primary Food Processing

Primary food processing involves the harvesting and initial conversion of plant and animal organisms into food and includes agricultural activities such as harvesting, slaughter, cleaning, sorting, and grading.

Proper allergen risk mitigation starts at this stage. The current enforcement system only tests terminated packaged foods and responds in a reactive manner with food recalls as the instrument to protect consumers. For some highly sensitized individuals a food recall is a measure that responds too late and which is incapable of preventing severe allergic reactions.

As an example, some fish allergic individuals have very specific sensitization for certain species of fish but could be tolerant to other fish species which could provide an opportunity to enrich the diet. The misidentification and therefore mislabelling of harvested fish species constitutes a potential risk of unintended exposure for such allergic consumers. Misidentification risks are of less concern when fish is grown and harvested in an aquaculture operation. In Ireland, some 25% cod and haddock products and as much as 82% smoked fish were found mislabelled using molecular biology techniques [13]. Similarly, some 75% of the fish sold in the United States of America as red snapper (the US Food and Drug Administration's legally designated common name for *Lutjanus campechanus*) belong to another species [14].

Agricultural activity presents its own challenges; nonallergenic crops contaminated with allergenic crops are an important risk to allergic consumers and can be compared to the historical contamination of wheat with the weed-plant purple cockle (*Agrostemma githago*) whose seeds are poisonous to the population at large; this contamination of the seeds carried over to the next planting season resulting in perpetuation or even amplification of the problem [15]. Certain contaminations of grains are particularly hard to detect due to the similarity of the kernels like soybean contamination of corn, or wheat in oats. Much of the cross-contact risk for plant foods is minimized by Good Agricultural Practice (GAP) but additional measures could be taken to protect allergic consumers.

Similar to the more widely known Good Manufacturing Practice (GMP), GAP is a collection of methods including record keeping, which is designed and implemented to achieve a particular purpose mainly quality preservation, but can also be extended to food security, food safety, sustainability, and ecology [16].

**Figure 1:**
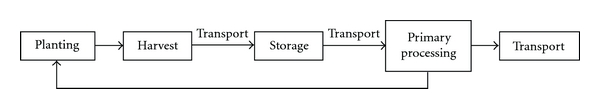
Schematic of the stages involved in food production and primary processing. Cross-contact with other crops, including allergenic organisms, can occur at any point in the process and can be magnified if harvested contaminated seeds are the primary material in the next planting season.

Figure 1 shows a general schematic of the agricultural processes used in seed-food production (e.g., cereals, oilseeds, and pulses). Cross-contact with other plant species can occur at any point during this process. After primary processing which includes general cleaning and sorting, seeds can be kept for the following season and replanted; or heat treated to stop enzymatic activity which can alter taste followed by transportation for further processing. Wagons, trucks, and bins (silos) previously used to transport and store other crops can easily hold remnants of the previous crop and contaminate newly harvested crop. Machinery used to harvest seeds (usually a combine harvester) and cleaning and sorting mills can also hold significant amounts of previously processed crops thus contaminating newly harvested crop. Farmers concerned about cross-contact can take additional measures. These include thoroughly cleaning harvest combines, trucks, and bins; using dedicated cleaning/sorting mills; procuring bare land around the planted plot; carefully documenting and planning crop rotation; obtaining fertilizer in bags rather than bulk format which are distributed in trucks or wagons which could have been used to hold crops beforehand. Most of these measures go beyond GAP but may be required if seeds are to be labelled as allergen-free.

Allergen contamination in finished prepackaged food products has been extensively studied and is the focus of most legislation. However, the contamination status of bulk food ingredients before and after primary food processing is often unknown.

Common agricultural practices include the use of green manure and cover crops to provide nutrient and minimize invasive weeds or earth erosion and crop rotation; leguminous plants are usually rotated (planted in alternating seasons) with other crops due to their soil nitrogen fixation abilities which lessen the need for fertilizers. Farmers generally follow a three-year rotation pattern of peanuts with cotton, corn or small grains planted on the same land in intervening years. The complete removal of the peanut plant at harvest diminishes the risk of cross-contact with other cultivated crops; also harvesting techniques and equipment are radically different between peanuts and grains, but even in the event of peanut contamination of other grains, the difference in size of the produce is large enough for the sorting/cleaning operation to be effective at removing contaminants. Besides peanuts, the tillage of cover and rotation crops eradicates most of the previously planted species, but does not eliminate the risk of cross-contact. A few of the plants turned into the earth will be able to grow back and contaminate the next crop at harvest, unless tillage is performed quite a few times enough to exhaust the plants' stored energy and/or badly injure the plant to cause its death.

Mustard seed is a relative of canola that has the advantage of being tolerant to drought, heat, and frost. It is an annual, cool-season crop that can be grown in a short growing season, commonly in rotation with cereal grains. The potential for mustard to contaminate grains like wheat, buckwheat, flax, and canola exists and, therefore, needs to be assessed.

Currently, there are several standards used when neat groats, kernels, or beans are sold. For example, the Codex standard for gluten-free foods specifies a maximum of 20 ppm of gluten; however, other Codex standards exist for “unprocessed” grains and pulses which establish a variable tolerance of 1 to 3% for contamination with extraneous matter and/or other grains. In the case of oats, as an example, this can be as high as 3% maximum edible grains other than oats. This tolerance represents an extremely high amount in terms of potential allergenic contamination since it allows up to 30 000 ppm (3%) of wheat, barley, and/or rye in oat kernels. Some currently accepted levels of contamination in various crops in different countries are provided in Table 2 [17–20].

The different levels of foreign material allowed in different crops (Table 2) are greatly influenced by the market. Higher levels of contamination are expected for lower crop grades; however, inherent technological challenges in the cleaning process also exist which helps to explain the differences across crops. Segregation of machinery and effectiveness of the cleaning milling operation is reflected in the lower limits for lentils. In cases such as sorghum, the lower economic importance for Canada is reflected in the lack of regulation.

### 2.2. Issues at Secondary Food Processing

Industrialized food production is a complex globalized endeavour with ingredient sourcing from many different parts of the world, tight schedules, and pressing requirements for very high productivity and profitability. As with any production operation, these systems are not always perfect. Some common production practices increase the risk of cross-contact (e.g., push-through uninterrupted production of different flavour ice creams; sharing of production equipment for manufacturing of foods with very different list of ingredients; or the indiscriminate use of rework in many food sectors including the bakery industry) [21].

**Figure 2:**
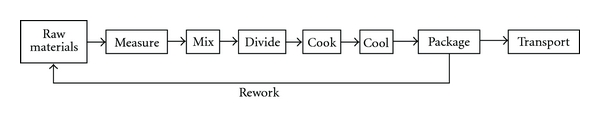
Example of rework in a food manufacturing process. Rework is the incorporation of preworked packaged food into new production batches as raw materials, but rework can be also derived from all intermediate steps before packaging. Rework is an important source of allergen cross-contact in the food manufacturing process.

Figure 2 represents a general schematic of the food manufacturing process showing rework as the incorporation of preworked packaged food into new production batches as raw materials. The risk, however, is that rework can be recuperated from all the intermediate steps of the process before packaging (i.e., after measuring, mixing, dividing, cooking, cooling, packaging, etc.). In certain industries, rework can go as far as the recycling of processed, packaged end-products which did not comply with quality controls for nonsafety-related specifications, such as appearance.

In multisector bakery products manufacturing, rework from pastry lines is occasionally incorporated into lower end bakery products regardless of the inclusion of allergenic ingredients in the dough which could result in the presence of hidden allergens and violation of local and sometimes international allergen labelling legislation.

Unrefined oils usually contain a higher amount of residual proteins from the starting raw material compared to purified oils; however, recently some refined oils were found to contain enough residual proteins to elicit IgE-mediated reactions in patients [22].

Another issue of concern is the contamination of food ingredients at source which could generate finished prepackaged foods containing ingredients not normally used as a typical ingredient of the food, such as wheat contaminated rolled oatmeal (gluten contamination). Gluten contamination of Canadian commercial oats was detected in 8 of 12 tested oats samples [23], and more recently 88% of samples on a larger Canadian survey was found contaminated by gluten [24]. Similarly, a study in the USA found 9 out of 12 samples of oats to be gluten contaminated [25]; another in Europe found 13% of oats products heavily contaminated with gluten with over 200 ppm [26].

For these reasons, HACCP programs in food manufacturing plants should include the analysis of critical control points for allergen contamination in order to effectively mitigate risks for consumers. Regular testing for allergens may be necessary as part of an allergen management plan at the manufacturing level in order to establish and monitor control limits with appropriate corrective actions to resolve deviations from normal acceptable levels. Thus, allergen testing tools that are simple to use and reliable are of paramount importance for the food industry.

Although testing methods for food allergens with excellent sensitivity and selectivity have been developed and commercialized, they are still subject to inaccuracies due to matrix and processing effects and stability issues [27, 28]. Conformational epitopes can be modified by processing or residence time within the food matrix [29], although linear epitopes will generally withstand denaturant conditions in the food. The need for processed allergen reference materials that can help in research and allergen surveillance in processed food products has been recognized and various research studies have shown that differences in food matrices can affect allergen recovery and protein structure which may alter immunodetection [30].

As many foods are likely to contain multiple allergens, there also continues to be a need for methods to detect the presence of multiple allergenic proteins. Simultaneous detection of food allergens in foodstuffs is possible by quantitative real-time polymerase chain reaction (qPCR), but this could become cumbersome and prone to unspecific DNA amplification when too many primers are involved; there are also matrix effects that can have an important impact on the technique therefore limiting a real multiplexing application of the method. Legitimate criticism has also been raised against the validity of DNA as a molecular marker to test the presence in the food of allergenic proteins or peptides, particularly after processing.

Recently, a combination technique has been developed for the recognition of multiple fish species parvalbumin. The detection is based on the hybridization of DNA probes on beads to amplified DNA of food samples [31].

Other recent developments in multiplexed detection of allergens have been made using beads-based immunoassays but the extended environmental testing is still limited to nonfood allergens: dust mite, cat, dog, rat, mouse, cockroach, and ragweed [32, 33]. Advances in mass spectrometry have permitted the recent multiplexing of food allergens detection, but the technique remains price-prohibiting and testing times could be long when a proteolysis step (sometimes overnight) is needed [34–36].

## 3. Food Ingredients, Allergenic Fractions, and Recognized Allergens

### 3.1. Peanuts

The seeds of the leguminous crop *Arachis hypogaea* are processed to obtain a limited number of food ingredients: peanut oil, peanut butter, and peanut flour. Peanut ingredients (butter and flour) were once used to increase protein content, flavour, and taste of foods until the risks of peanut allergy were acknowledged; today, the use of these ingredients still remains popular in the food industry. Besides peanut ingredients, whole roasted peanuts also find wide use in confectionary products alongside tree nuts.

Studies carried out in Montreal, Canada found 1.5% of young school children (up to grade 3) were sensitized to peanuts [37]. Eleven relevant allergens of peanut has been identified *Ara h*1 to *Ara h*9 [38–43] plus two peanut oleosins designated *Ara h*10 and *Ara h*11 [44]. Traditionally *Ara h*1 a protein of 63 kDa and *Ara h*2 a 17–19 kDa doublet, have been designated the major peanut allergens based on the frequent and intense binding of IgE to these proteins on immunoblots with sera from peanut-allergic patients. Recently, *Ara h*2 has been reported as the most potent allergen from peanuts [45]; additionally, *Ara h*2 shows cross-reactivity to almond and Brazil nut [46]. Another cross-reactivity between *Ara h*8 and *Bet v*1 was observed; this is of special importance to Europeans given the abundance of birch trees in the region [47, 48].

Detection of peanuts allergens has been investigated using qPCR and ELISA with limits of detection (LODs) as low as 0.5 ng per mL [49, 50]. Also, combination techniques like liquid chromatography coupled with immunomagnetic beads has been investigated [51].

### 3.2. Soybeans

Botanically, soy (*Glycine max*) is a legume but similar to peanuts it is considered an oilseed from the a food technology point of view since it is not cultivated to be consumed in the pod like snow peas or for the dry grain like red kidney beans. Besides the numerous traditional dishes prepared with soy in Asia, for example, tofu, tempeh, soysauce, soymilk, and miso; soybean ingredients have been developed for a great variety of mainstream food uses and include soybean oil, soy flour (min. 50% protein), flakes, grits, soy protein concentrate (65–85% protein), soy protein isolate (>85% protein), soybean lecithin, and soybean fibre. A comprehensive list of primary and secondary ingredients from soy has been reported elsewhere [52].

Six different allergens in soybean have been designated: *Gly m*1 and *Gly m*2 are aeroallergens responsible for asthma reactivity [53]; *Gly m*3 is a 14 kDa profilin that shows cross-reactivity with birch pollen profilin *Bet v*2 [54]; *Gly m*4 a disease resistance response protein of 17 kDa is present in soy products in variable quantities and it is also related to birch pollen *Bet v*1 [55, 56]; the two major soybean storage proteins are also allergenic, *β*-conglycinin a vicilin, 7S globulin denominated *Gly m*5 of 140–170 kDa; glycinin an 11S globulin of 320–360 kDa denominated *Gly m*6 [57].

Detection methods for soybeans in food include ELISA-based methods [58], PCR, and qPCR-based methods [59], and combination methods like aggregation immunoassay involving the use of gold nanoparticles coupled with light scattering detection [60]. Detection and quantification methods for soybean allergens also depend on the protein extraction procedure from the food matrix. Specific extraction methods have been developed and standardized [61].

### 3.3. Tree Nuts

Tree nuts are the fruits or seeds of various tree species from the orders Rosales, Sapindales, Fagales, Ericales, Proteales, and Pinales, contained within a hard shell. These species do not form a taxonomic group but rather a functional or agronomic one. Tree nuts are consumed as mixed nuts usually roasted, or used in specialty bakery, pastry, and confections.

Allergen cross-reactivity is frequent and extensive within this group; pollinosis has also been observed persistently. In addition to serious and acute reactions including systemic reactions to tree nuts, a commonly observed reaction is oral allergy syndrome (OAS). OAS is characterized by itching or burning of the mouth, lips, tongue, and/or throat, with concomitant local inflammation [62].

Simultaneous reactions to tree nuts were observed in 12 of 62 patients studied, with the most common allergic reaction to Brazil nut plus other nuts. Also, allergy to peanuts plus other tree nuts was observed in 12 other patients [63].

Allergens from cashew (*Anacardium occidentale*) include the major allergen *Ana o*1, a 7S vicilin-like protein; a homotrimer of 45 kDa subunits. Cashew and peanut vicilins do not share linear epitopes [64]. *Ana o*2 of 55 kDa, encode for a member of the legumin family (an 11S globulin) of seed storage proteins [65]. *Ana o*3 of 14 kDa is a 2S albumin [66].

Pistachio (*Pistacia vera*) allergens *Pis v*1 (7 kDa) and *Pis v*2 (32 kDa), belong to the 2S albumin and 11S globulin family, respectively [67]; *Pis v*3 of 55 kDa is a 7S vicilin-like protein [68]; *Pis v*4 a 23 kDa manganese superoxide dismutase-like protein [69]; a minor pistachio allergen *Pis v*5 is an 11S globulin precursor peptide [70].

Walnut (mostly *Juglans regia* but also* J. nigra*): *Jug r*1 a 2S albumin [71]; *Jug r*2 a 7S vicilin-like globulin [72]; *Jug r*3 a 9 kDa lipid transfer protein (LTP) [73]; *Jug r*4 an 11S legumin-like globulin [74].

Hazelnut (*Corylus avellana*): *Cor a* 1.04 is the major food allergen from hazelnut and it is closely related to birch pollen allergen *Bet v*1, but much less related with only 63% sequence homology to hazel pollen allergen *Cor a* 1 [75]; less prevalent *Cor a*2 is a profilin homologous to *Bet v* 2 [76]; *Cor a*8 and *Cor a*9 are, respectively, an LTP and 11S globulin-like seed storage protein identified as a legumin, these two minor allergens are involved in life-threatening reactions to hazelnut [77]; *Cor a*11 a vicilin-like 7S is a minor hazelnut allergen [78]; two oleosin isoforms of 17 and 14–16 kDa, now designated *Cor a*12 and *Cor a*13, were identified as new allergens in hazelnut [79]; *Cor a*14 is a 2S albumin of 15-16 kDa from hazelnut [80].

Almond (*Prunus dulcis*): almond major protein or amandin designated *Pru du*6 is the major seed storage protein of almond with 360 kDa an 11S globulin legumin-like protein [81]; *Pru du*4 a profilin, cross-reactive to ryegrass pollen profilins [82]; *Pru du*3 a nonspecific LTP of 9 kDa [83]; *Pru du* 5a 10 kDa 60s acidic ribosomal protein [84].

Brazil nut (*Bertholletia excelsa*): *Ber e*1 is a 9 kDa 2S seed storage albumin [85], and *Ber e*2 is a 29 kDa 11S globulin legumin-like protein [86].

Macadamia nut (*Macadamia integrifolia*, *M. tetraphylla*, and their hybrids): although not as commonly consumed as other tree nuts, macadamia can occasionally cause serious allergic reactions like angioedema and dyspnoea [87, 88]. A previous case of anaphylaxis showed strong serum IgE binding to a protein of 17.4 kDa from both raw and roasted extracts [89]. There are no designated allergens for macadamia nut to date.

There are only two recognized allergens of pecan (*Carya illinoinensis*). *Car i*1 is a 16 kDa 2S albumin seed storage protein [90], and *Car i*4 is a legumin 11S seed storage protein, hexameric with 55.4 kDa per monomer [91].

There are no designated allergens for pine nut (*Pinus* spp.) to date; although a 17 kDa allergenic protein has been detected [92]. Allergic reactions to pine nuts have been reported and include skin reactions, angioedema, hypotension, and anaphylaxis among others [93–96].

There are many protocols for detection of tree nuts in food. Some examples of analytical techniques include ELISA-based methods for detection of walnut [97], pecan [98], almond [99], and Brazil nut [100]; qPCR for detection of macadamia nut [101], hazelnut [102], pecan [103], and cashew [104]; time-resolved fluoroimmunoassay for hazelnut [105]. Simultaneous detection of multiple tree nuts is possible with qPCR-based methodology [106].

### 3.4. Sesame Seeds

*Sesamum indicum* seeds are mainly used whole dried or toasted for culinary purposes, and sesame oil is used in salad dressing in Oriental, Chinese, and South American cuisines. The production and use of sesame oil is restricted to Mid and Far East and used primarily as a flavouring agent. Sesame seeds are a common sight as garnish of hamburgers' buns (breads), certain confectionary products, crackers, chips, vegetable patties (burgers), and oriental specialities.

Research on sesame seed allergens is recent and has allowed the identification of multiple important allergenic fractions: *Ses i*1 a 9 kDa, 2S albumin [107]; *Ses i*2 another 2S albumin of 7 kDa; *Ses i*3 a 45 kDa, 7S vicilin-type globulin [108]; *Ses i*4 and *Ses i*5 are oleosins with 17 and 15 kDa, respectively [109]; two minor allergens *Ses i*6 and *Ses i*7 were identified as 11S globulins with 52 and 57 kDa respectively [110].

Detection of sesame allergens can be accomplished by qPCR assays [111, 112] or ELISA [113] with LOQ as low as 49 *μ*g per g of sesame flour in food.

### 3.5. Wheat

Wheat (*Triticum* spp.) belongs to the Triticeae tribe within the Gramineae family of grasses. Of immense economic importance, wheat is the third grain grown globally after corn and rice. In the five years period from 2004 to 2008, the average world production of corn was 752 million tonnes, rice 645 million tonnes, and wheat 633 million tonnes; but adding up the production of wheat, barley, rye, and triticale (hybrid of wheat and rye) the figure goes up to 806 million tonnes which makes this group the largest cereal produced worldwide [114]. Many foods are made with wheat and its derived ingredients: flour, starch, hydrolyzed wheat protein, and so forth; therefore an avoidance diet for sensitized patients is a difficult proposition.

Food allergens identified in wheat include *Tri a*12 a profilin of 14 kDa [115]; *Tri a*14 a nonspecific LTP1 of 9 kDa [116]; *Tri a*18 agglutinin isolectin 1 [117]; *Tri a*19 omega-5 gliadin, a seed storage protein of 65 kDa [118, 119]; *Tri a*25 thioredoxin [120]; *Tri a*26 a glutenin of 88 kDa [121].

Aside from the IgE-mediated allergic response that wheat and related grasses can create in sensitized individuals, the importance of including wheat and other sources of gluten (or related proteins) as a priority allergen in the *Codex Alimentarius* derives from the greater and growing prevalence of celiac disease among the world population. Gluten sources have to be declared on packaging in many countries when a food contains gluten protein or modified gluten protein.

For celiac individuals it is the gluten protein or more importantly the prolamins contained in oats, barley, rye, triticale, or wheat, including kamut or spelt which causes the cell-mediated immunologic reaction with the consequent abdominal and nonabdominal symptoms. Recent studies have suggested that the prolamin from oats (avenin) is not toxic to celiacs [122–125], but the problem appears to reside in the contamination of oats by wheat, barley, or rye. Gluten contamination of commercial oats' products has been detected in various studies and therefore deserves further investigation and surveillance [24, 25].

### 3.6. Mustard Seeds

Canada was the top world exporter of mustard seeds in the five-year period of 2004 to 2008 [114]. There are three industrial cultivars of mustard: black mustard (*Brassica nigra*), oriental mustard (*B. juncea*), and yellow, also referred to as white mustard (*Sinapis alba* or* B. hirta*). European regulations include mustard as an allergen to be declared on food labels. Mustard has also been recently added to the Canadian list of priority allergens after extensive public consultation and review of the literature. Mustard seeds are principally used in the preparation of mustard condiments for which all three cultivars have specific uses, although *S. alba* seeds are the most frequently employed to produce the common yellow mustard condiment. Out of the three species, yellow seeds are the mildest also showing the lowest oil content. Oriental mustard seed is often used to produce spicy cooking oils utilized in traditional Asian cuisine. Mustard seeds are also used whole in spice blends or ground into flour which has multiple uses in processed foods like mayonnaise, salad dressings, soups, and processed meats for its taste, but also for emulsification and water holding capacity properties.

Mustard allergy accounts for 1.1% of food allergies in French children [126, 127]. The most predominant allergenic protein of yellow mustard, *Sin a*1, is a 2S seed storage albumin, a compact molecule with molecular mass of 14.18 kDa; this thermostable protein is resistant to *in vitro* digestion by trypsin and degradation by other proteolytic enzymes [128]. The principal allergen of *B. juncea* seed is *Bra j*1 with a structure very close to *Sin a*1 [129]. Another storage protein the 11S globulin *Sin a*2 of 51 kDa has recently been identified as an important allergen [130, 131]. A couple of allergens derived from nonstorage seed proteins have also been identified (*Sin a*3 a nonspecific LTP of 12.3 kDa and *Sin a*4 a profilin of 13-14 kDa) which show IgE cross-reactivity with peach and melon fruits, respectively [132].

Quantitative detection of mustard allergens in food can be accomplished by sandwich-type ELISA with LODs as low as 1 *μ*g of ground whole mustard seeds per mL [133, 134] or qPCR [111].

### 3.7. Milk

Milk is defined as the mammary glands' secretion of many animal species mostly cattle, sheep, goats, and buffalo. Milk is widely used as food ingredient after standardization, homogenization, and pasteurization. Many other food ingredients are derived from milk including cream, butter, cheese, and protein derivatives such as caseinates, whey protein, protein hydrolysates, and lactose. Due to the diverse list of ingredients derived from milk and the use of milk itself in a multitude of foods, it is a difficult allergen to avoid.

Cow's (*Bos taurus domesticus*) milk allergy is well documented and extensively studied. *α*_S1_- and *β*-casein fractions from the milk coagulum and *β*-lactoglobulin from the lactoserum fraction are important allergens; in fact, all milk protein fractions display some degree of antigenicity with a multitude of conformational as well as linear epitopes [135, 136]. Formally designated allergens from milk are denominated *Bos d*4 to *Bos d*8 which refer respectively to *α*-lactalbumin, *β*-lactoglobulin, serum albumin, immunoglobulin, and caseins. Polysensitization and cross-reactivity occurs between different milk protein fractions and among milk from different species making the selection for a cow's milk substitute among milk from other ungulates a very difficult task [136–138].

One popular approach for production of hypoallergenic baby formula is the use of partially hydrolyzed whey proteins. In these formulations the allergens of the casein fraction from milk are not present, and the allergens from the whey proteins are modified by hydrolysis, diminishing conformational epitopes, although linear epitopes could still remain. The degree of hydrolyzation should be controlled as extensive hydrolyzation creates bitter peptides. The use of partly-digested milk protein-based baby formulas do not eliminate all allergens, therefore it is usually advised as a preventative measure when there is a family history of atopy. Other formulations (soybeans or rice based) should be sought when milk allergy is confirmed for the infant.

As there are no cures for food allergies at the present time, complete avoidance of the allergenic food is the commonly prescribed therapy. In practical terms a zero-tolerance limit presents many challenges and allergen occurrence thresholds for enforcement agencies are often established based on detection and quantification limits of analytical techniques. There are several methods developed to detect and quantify the different allergens in milk [139]. Many ELISA-based methods have been developed and some are commercially available. Recent combination methods have been investigated based on different techniques such as liquid chromatography with mass spectrometry detection [140], specialized extraction coupled with ELISA detection [141], and surface plasmon resonance-based immunosensors [142], among others.

### 3.8. Eggs

Chicken (*Gallus gallus domesticus*) eggs are a very common food ingredient. They are used whole or as separated egg white and egg yolk. Eggs are a very important food ingredient from the technological stand point, since emulsifiers are found in egg yolk and foaming agents in egg white; although some of these functionalities can be simulated by other ingredients such as plant-derived emulsifiers and plant or micro-organism extracted gums, there is a price penalty.

Egg allergy is common among children, with prevalence calculated at 1.6% at 2.5 years of age [143]. The condition can be reversed, with as many as 11–50% of infants developing tolerance to eggs by age 4–4.5, and 82% by age 16 [144, 145]. The level of IgE to egg has been reported as a good predictor of clinical symptoms, and a level of ≥50 kU/L egg IgE as an indication of persistent egg allergy that will unlikely resolve before age 18 [144]. New oral immunotherapy has been successfully tested with potential for tolerance development [146]. A peculiar phenomenon of documented cross-reactivity is called the bird-egg syndrome [147], where sensitization for egg yolk livetins occurs via bird's aeroallergens [148].

Four allergens from hen's egg white have been documented *Gal d*1 to *Gal d*4 which are, respectively, ovomucoid, ovalbumin, ovotransferrin, and lysozyme. Additionally, two allergens from egg yolk have been characterized, *Gal d*5 or *α*-livetin [149]; YGP42 protein, a fragment of the vitellogenin-1 precursor denominated *Gal d*6 [150]. All these proteins except for lysozyme exhibit different degrees of polymorphism and glycosylation [151]. 

Testing methodology for the presence of eggs in foods include ELISA-based tests [152] and qPCR [153].

### 3.9. Seafood

This group of allergenic foods is composed of crustaceans, shellfish, and fish, therefore many animal species comprising several allergenic proteins are included. Given the dominant flavour of this food group, seafood is usually not found as a contaminant of other food groups; in contrast, Asian cuisine makes intense use of seafood stock and fermented fish sauces as base flavour for many dishes.

Of all the allergenic fractions of seafood, *β*-parvalbumin stands out as a major allergen; this protein has been characterized and immunologically assessed in many fish species: Atlantic herring (*Clupea harengus*), Pacific pilchard (*Sardinops sagax*), Baltic cod (*Gadus callarias*), yellowfin tuna (*Thunnus albacares*), swordfish (*Xiphias gladius*), Atlantic salmon (*Salmo salar*), and ocean perch (*Sebastes marinus*) [154]. Allergic individuals usually avoid all species of fish while some people may tolerate a few, which is an indication of specific epitopes per fish species allergen.

Since the classical work of Shanti et al. [155] describing the allergenic characteristics of shrimp's (*Penaeus indicus*) major muscle protein tropomyosin, now officially denominated *Pen i*1, many food tropomyosins from other Decapoda species have been characterized and recognized in crab (*Charybdis feriatus*), shrimp (*Metapenaeus ensis* and *Penaeus aztecus*), white shrimp (*Litopenaeus vannamei*), North Sea shrimp (*Crangon crangon*), black tiger shrimp (*Penaeus monodon*), american lobster (*Homarus americanus*), spiny lobster (*Panulirus stimpsoni*), and also recognized in squid (*Todarodes pacificus*) and *Anisakis simplex* which is a nematode parasitic of marine mammals, crustacean, and fish. Tropomyosin can cause anaphylaxis in sensitized consumers who consume raw or processed seafood and fish [154].

### 3.10. Sulphites

Sulphites or sulfites are widely used food preservatives, employed to extend shelf life of foods and maintain food colour due to its antioxidant properties that prevent enzymatic and nonenzymatic browning. Its antimicrobial properties are also well known and historically employed in the food industry.

Sensitivity to sulphites is not an allergy *per se* but rather an adverse acute reaction to this inorganic substance, although IgE-mediated responses have been identified [156]. There are several compounds used in the food industry from which the water-soluble sulphite anion SO_3_^2−^ is derived: sulphur dioxide, sodium sulphite, and potassium and sodium salts of bisulphite and metabisulphite. Incorporation of sulphites in recipes or its natural occurrence in excess of 10 ppm has to be declared in Australian, Canadian, New Zealand, and European food labels. USA standard labelling requires its declaration when present in excess of 10 ppm, but it is not part of the USA priority allergens list. 

The amount of this preservative in foods markedly varies from around 10 ppm in frozen dough, corn syrup, and jellies, to up to 60 ppm in fresh shrimp, pickles, and fresh mushrooms, to up to 100 ppm in dried potatoes, wine, vinegar, and maraschino cherries, and up to 1000 ppm and beyond in dried fruit; lemon, lime, grape, and sauerkraut juice; some retail made-in-place fresh sauces [2, 157]. There is a compelling body of knowledge which indicates exacerbation of symptoms (bronchospasms) in sulphite-sensitive asthmatic individuals after ingestion of sulphites [157–162], although this has recently been challenged for sulphite-containing wines [163]. 

Japanese legislation requires sulphites to be declared as additives (bleaching agents) and allows from 30 ppm in squeezed fruit juice, 1500 ppm for raisins, 2000 ppm in dried fruits, up to 5000 ppm in kanpyo (dried gourd strips) [164].

Sulphites content determination in food is traditionally accomplished by the Monier-Williams distillation method [165]. Fast detection methods have also been developed like an enzyme electrode assay [166], flow injection analysis with voltametric detection system [167], ion chromatography with electrochemical detection [168], HPLC-fluorescence spectrometry method [169], ion-exchange chromatography with conductivity detection [170], and many others.

## 4. Concluding Remarks

Priority allergens lists are in constant review and prone to modifications to adapt them to regional epidemiological changes in allergic subpopulations. However, it is difficult to determine the accurate populations' prevalence of food allergies, and comparisons are most of the time invalid partially because of differences in methodologies and general testing criteria. Accurate food allergy incidence figures are elusive and cross-reactivity frequency estimations are even more obscure. Nonetheless, the obligation to protect the allergic public has been recognized by governments and international entities.

There is a need to apply a bottom-up approach to allergen risk management in the food manufacturing process starting from primary food processing practices in order to ensure greater food safety for allergic consumers. Assessment of the allergen contamination status of food ingredients at the primary processing level is of vital importance as it will help in the development of improved integrated solutions for allergen risk mitigation and in the establishment of a proactive food surveillance system.

For the food manufacturing industry the “clean-label” trend which calls for minimization of the number of ingredients in recipes has had a positive impact on production costs by consolidating and simplifying the sourcing of ingredients. This in turn may help in minimizing cross-contact of ingredients; however, the allergenic load in these raw materials after primary processing needs to be assured.

## Figures and Tables

**Table 1:** Lowest amount of allergenic food to elicit an observed objective adverse effect (LOAEL) and limit of detection of contaminants (allergens) in foods.

Contaminant	LOAEL (mg of protein)	Method of detection	LOD (ppm)
Peanuts	0.25–10	ELISA	0.1
Soybeans	88–522	ELISA	0.016
Tree nuts	0.02–7.5	ELISA	0.06
Sesame seeds	30	ELISA	0.2
Gluten	20–100	ELISA	0.6
Mustard seeds	1–936	ELISA	1
Milk	0.36–3.6	ELISA	0.00004
Egg	0.13–1.0	ELISA	0.05
Seafood	1–100	ELISA	0.0009
Sulphites		Monier-Williams distillation	10

Data from multiple sources [165, 171–175]; LOAEL—lowest observed adverse effect level; LOD—limit of detection; empty cell means no data was found.

**Table 2:** Current maximum accepted levels of foreign material allowed in various crops in Canada, USA, and Europe.

Crops*∖*grade	Foreign material allowed (%)										
	Canada (CE, CW)					US					EU
	1	2	3	4	5	1	2	3	4	5	
Oats	1, 0.75 w 1, 0.75 b NS, 1 c	2, 1.5 w 2, 1.5 b NS, 2 c	6, 3 w 6, 3 b NS, 3 c	14, 8 w 14, 8 b NS, 8 c	NA	2.0	3.0	4.0	5.0	NA	2
Corn	2, 2	3, 3	5, 5	7, 7	12, 12	2.0	3.0	4.0	5.0	7.0	5
Buckwheat	1	2.5	5	NA	NA						
Sorghum						1.0	2.0	3.0	4.0	NA	5
Soybean	1	2	3	5	8	1.0	2.0	3.0	5.0	NA	
Lentils	0.2	0.5	1	NA	NA	0.2	0.5	0.5	NA	NA	

CE—Canada East; CW—Canada West; w—wheat; b—barley; c—other cereals; NA—not applicable (grade does not exists for the given crop); NS—not specified; empty cell means no data was found.
